# Spatial accessibility to healthcare services in Shenzhen, China: improving the multi-modal two-step floating catchment area method by estimating travel time via online map APIs

**DOI:** 10.1186/s12913-018-3132-8

**Published:** 2018-05-09

**Authors:** Zhuolin Tao, Zaoxing Yao, Hui Kong, Fei Duan, Guicai Li

**Affiliations:** 1 School of Urban Planning and Design, Peking University, Shenzhen, 518055 Guangdong China; 2 Economics and Business Administration, Chongqing University, Chongqing, 400030 China; 3 Department of Geography, The Ohio State University, Columbus, OH 43210 USA

**Keywords:** Healthcare accessibility, Multi-modal, 2SFCA, Online map API, Shenzhen

## Abstract

**Background:**

Shenzhen has rapidly grown into a megacity in the recent decades. It is a challenging task for the Shenzhen government to provide sufficient healthcare services. The spatial configuration of healthcare services can influence the convenience for the consumers to obtain healthcare services. Spatial accessibility has been widely adopted as a scientific measurement for evaluating the rationality of the spatial configuration of healthcare services.

**Methods:**

The multi-modal two-step floating catchment area (2SFCA) method is an important advance in the field of healthcare accessibility modelling, which enables the simultaneous assessment of spatial accessibility via multiple transport modes. This study further develops the multi-modal 2SFCA method by introducing online map APIs to improve the estimation of travel time by public transit or by car respectively.

**Results:**

As the results show, the distribution of healthcare accessibility by multi-modal 2SFCA shows significant spatial disparity. Moreover, by dividing the multi-modal accessibility into car-mode and transit-mode accessibility, this study discovers that the transit-mode subgroup is disadvantaged in the competition for healthcare services with the car-mode subgroup. The disparity in transit-mode accessibility is the main reason of the uneven pattern of healthcare accessibility in Shenzhen.

**Conclusions:**

The findings suggest improving the public transit conditions for accessing healthcare services to reduce the disparity of healthcare accessibility. More healthcare services should be allocated in the eastern and western Shenzhen, especially sub-districts in Dapeng District and western Bao’an District. As these findings cannot be drawn by the traditional single-modal 2SFCA method, the advantage of the multi-modal 2SFCA method is significant to both healthcare studies and healthcare system planning.

## Background

Healthcare service is essential in modern society, and is closely related to the overall health level of public. The People’s Republic of China issued the “Healthy China 2030 Planning Outline” in October 2016, which reveals the concerns in the health field at the national level. As one of the special economic zones in China, Shenzhen has rapidly grown into a megacity with more than 10 million population in the recent decades. As a result, it is a challenging task for the Shenzhen government to provide sufficient healthcare services for such an enormous population.

The spatial configuration of healthcare services can influence the convenience for the consumers to obtain healthcare services and the utilization of healthcare resources. An unreasonable distribution of healthcare services is usually considered as a cause of inequality in health outcomes [1]. Evidence from previous studies have shown that the access to healthcare services is an essential factor that will significantly influence health conditions [2, 3]. Therefore, minimizing inequity in accessibility to healthcare services is widely considered as an important goal in health-related policy making. Accessibility to healthcare services can be divided into spatial and nonspatial dimension: spatial accessibility (spatial dimension) emphasizes the spatial separation between supply and demand as a barrier or a facilitator, while nonspatial accessibility (nonspatial dimension) focuses on nongeographic factors [4–6]. Spatial accessibility has been widely adopted as a scientific measurement for the opportunities of obtaining services and the geographic disparity of such opportunities, which can be used to quantitatively assess the spatial configuration of healthcare services [1, 4].

Various methods have been developed for measuring spatial accessibility to public services especially healthcare services, including simple indices such as the supply-to-demand ratio and distance to the closest facility, and more complex methods such as the kernel density, gravity model and two-step floating catchment area method [4, 5]. Among these methods, the two-step floating catchment area (2SFCA) method is most commonly-used partially because of its advantage in understandability and operability [6]. 2SFCA emphasizes both the spatial impedance between demand and supply as well as the availability of services (i.e. the amount of supply that is available to a population group), making it a relatively comprehensive and advanced measurement [4–6]. Moreover, various improvements of 2SFCA have been proposed by recent studies, further enhancing its theoretical foundation and applicability [7–9].

Some studies have implemented 2SFCA method or its variants to measure spatial accessibility to healthcare services in Shenzhen. For example, Tong and Chen evaluated the accessibility to healthcare facilities in Shenzhen using the original 2SFCA method, and discovered significant disparity in healthcare accessibility [10]. Cheng et al. [11] measured accessibility to high-level hospitals in Shenzhen using the Kernel Density 2SFCA method [12, 13], which is an improvement of 2SFCA with an additional distance decay function. However, studies on healthcare accessibility in Shenzhen are still limited, and some advanced improvements of 2SFCA method have not been utilized for healthcare accessibility analyzing in Shenzhen.

An important branch of the improvements of 2SFCA is to consider multiple transport modes in accessibility analysis. Some studies have made attempts from this aspect. Dony et al. calculated and compared accessibility to parks using four transport modes (bicycling, driving, public transit, and walking) respectively [14]. Similarly, Cheng et al. calculated spatial accessibility to hospitals for both car and public transit [11]. However, in the above two studies, accessibility for different transport modes are calculated separately. As a result, the competition for healthcare services between populations with different transport modes (by car or by public transit) is neglected. The multi-modal 2SFCA method [15, 16] assesses the spatial accessibility via multiple transport modes simultaneously, which can provide more accurate measurement of spatial accessibility in a multi-modal context. As the first version of multi-modal 2SFCA, Mao and Nekorchuk’s model [15] has some shortcomings (see Section 3 for details). Langford et al. [16] proposed an improved version of multi-modal 2SFCA and overcame these shortcomings.

However, applications of multi-modal 2SFCA are still quite limited. One possible reason is that data of transport networks for estimating travel time by each mode are unavailable or outdated in many cases. Moreover, traditional estimation of travel time based on transport network dataset needs to set driving speed parameters, which is somewhat arbitrary in practice. To address these problems, this study proposes to utilize online map API (Application Programming Interface) to estimate travel time via multiple transport modes. Online map APIs are usually open for personal researchers. In this way, researchers can make use of the dynamically updated transport network data and the routing rules maintained by map developers to obtain a reliable estimation of travel time [17]. Online map APIs can improve the estimation of travel time for the multi-modal 2SFCA method and thus can promote its applications.

In this study, the multi-modal 2SFCA method will be applied to measure healthcare accessibility in Shenzhen, China, and online map APIs will be utilized to improve the travel time estimation of this method. As Baidu Map (similar to Google Map) is one of the most popular online maps in China, two Baidu Map APIs, the Transit Searching API and the Driving Searching API, are utilized to estimate travel time by public transit or by driving car respectively. Based on the results, regions in shortage of healthcare services can be identified, which can provide knowledge-based reference to the spatial configuration of healthcare services in Shenzhen.

The remainder of the paper is structured as follows. Section 2 describes the study area and data sources of population and healthcare facilities. In Section 3, the multi-modal 2SFCA method is developed based on the generalized 2SFCA framework. Estimation of travel time by different modes and setting of parameters are also presented in this section. Section 4 analyzes the results, by comparing multi-modal accessibility with traditional single-modal accessibility and comparing accessibility of subgroups with different modes. Finally, a brief summary is presented in Section 5.

## Study area and data

**Fig. 1:**
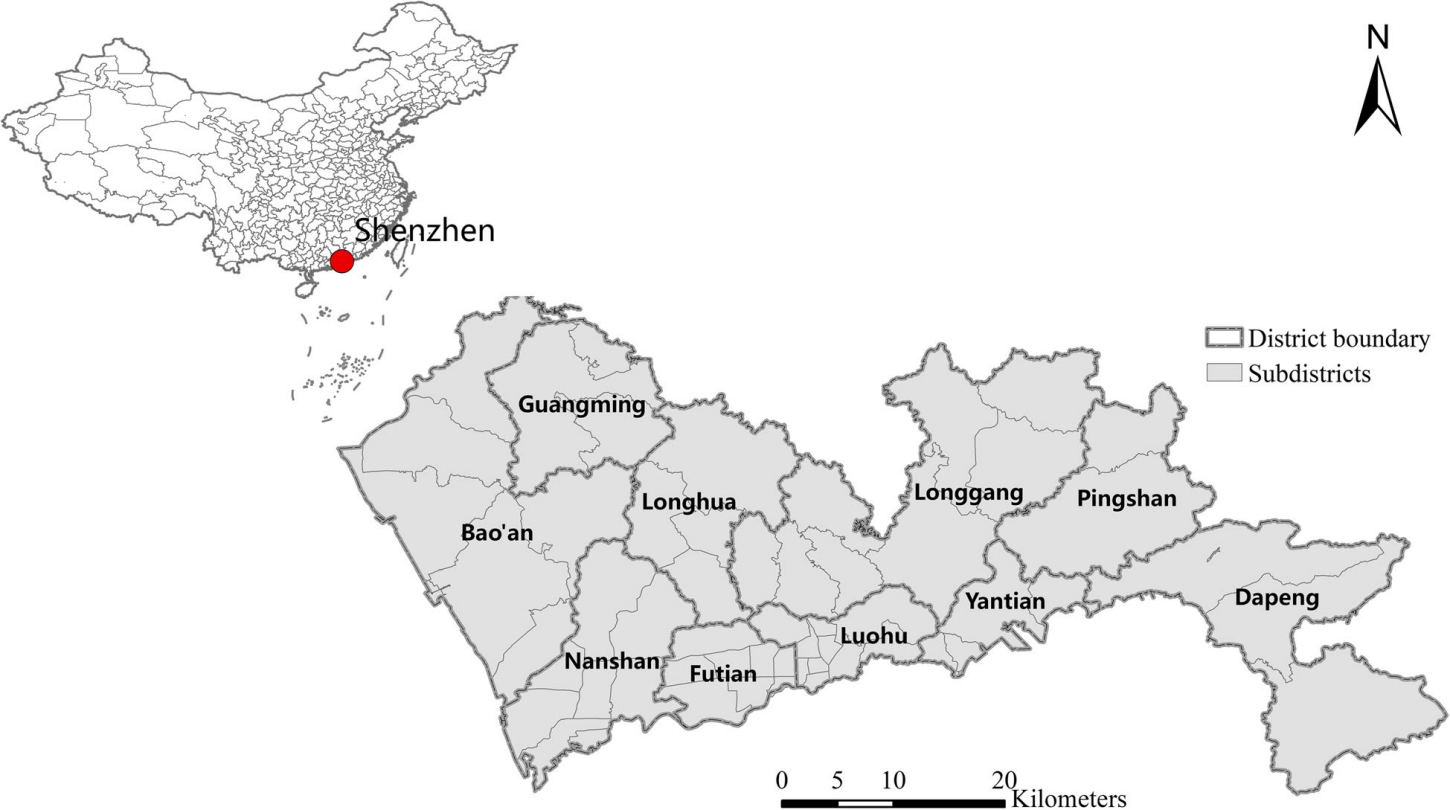
Location and administrative divisions of Shenzhen

Shenzhen, the study area, is located in Guangdong Province, south China (Fig. 1). It is one of the main cities in the Pearl River Delta, which is one of the most developed regions in China. Since the reform and opening-up of China in the 1980s, Shenzhen has experienced miraculous rapid growth for more than 30 years. Shenzhen has grown into a megacity with 10.78 million permanent residents and an administrative area of 1997 km^2^. There are 10 administrative districts in Shenzhen, including Luohu, Futian, Nanshan, Yantian, Bao’an, Longgang, Guangming, Longhua, Pingshan, and Dapeng, and 55 subdistrict units (Fig. 1). Luohu, Futian and Nanshan are the most developed and populous districts in Shenzhen.

The population data at the sub-district level used in this study comes from the 6th population census data of China at 2010, which is the latest official population census data at the sub-district level. Due to the lack of population data at a finer scale, the geometry centroids of sub-districts are used to denote the demand nodes. The total population of Shenzhen in 2010 is 10.36 million.

**Fig. 2:**
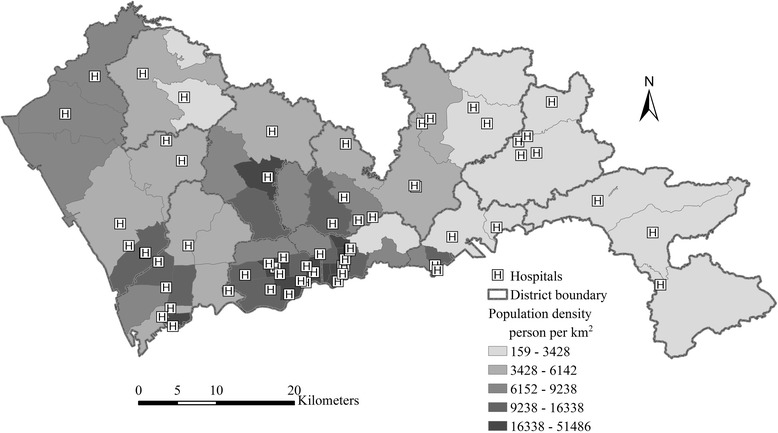
Distribution of population and hospitals in Shenzhen

List and addresses of healthcare facilities in Shenzhen are obtained from the official website of Shenzhen government (http://www.sz.gov.cn/ylwslyfwzt/kbjy/yljg/). The latest time of visiting the website was April 13, 2017. Only the hospitals listed on the above website are included in our analysis. The community healthcare centers are not considered, since they are usually small-scaled and dispersed in the space, and their service capacities are unknown. As shown in Fig. 2, there are 62 hospitals in Shenzhen. The number of physicians is used to denote the capacity of each hospital. There are 14,929 physicians in total.

## Methods

### The generalized 2SFCA framework

The 2SFCA method measures spatial accessibility by two steps. In the first step, it searches all demand nodes within the catchment area of each facility, then calculates the supply-to-demand ratio for each facility, i.e. the average supply per potential service user. In the second step, it adds up the supply-to-demand ratios of all facilities located within the catchment area of each demand node. The sum of supply-to-demand ratios for each demand node is its spatial accessibility score.

The original 2SFCA method adopts a dichotomous distance decay function [18], which assumes services to be equally accessible within the catchment area but completely inaccessible outside the catchment area. This assumption is usually considered as a disadvantage of the original 2SFCA method [19]. To overcome this disadvantage, several improvements of 2SFCA have introduced additional distance decay functions into the original 2SFCA method, including a multi-stage discrete function in the Enhanced 2SFCA [20], the Gaussian function [12], the Kernel density function [21], and the inverse power function [22]. These improvements can be unified by a generalized 2SFCA framework developed by Wang [1]:

1 $$ {A}_i={\sum}_j\frac{S_jf\left({d}_{ij}\right)}{\sum_k{D}_kf\left({d}_{kj}\right)} $$

where *A*_*i*_ is the accessibility at demand node *i*, *S*_*j*_ is the capacity of supply at location *j*, *D*_*k*_ is the demand amount, *d*_*ij*_(*d*_*kj*_) is the distance or travel time between *i*(*k*) and *j*, *f* is a general distance decay function. The distance decay function *f* can take various forms, as mentioned above. In this study, the Gaussian function is adopted as the distance-decay function, since it decreases slowly where the travel time is close to zero or close to the catchment area size. The distance decay function *f* can be written as [12, 13]:

2 $$ f\left({d}_{ij}\right)=\left\{\begin{array}{c}\frac{e^{-1/2\times {\left({d}_{ij}/{d}_0\right)}^2-}{e}^{-1/2}}{1-{e}^{-1/2}},{d}_{ij}\le {d}_0\\ {}\kern2.5em 0,\kern1.75em {d}_{ij}>{d}_0\end{array}\right. $$

where *d*_0_ is the size of catchment area.

### The multi-modal 2SFCA method

Most existing applications of 2SFCA methods only consider a single transport mode, typically by car [15]. These studies neglect the fact that demanders may travel to facilities (e.g. healthcare facilities) via various transportation modes, such as by private car, taxi, subway or bus. Particularly, for disadvantaged (e.g. low-income) populations without private cars, public transit may be the major transportation mode. Therefore, it is not sufficient if only a single transport mode is considered in spatial accessibility.

As was mentioned in the first section, two previous studies have attempted to propose multi-modal 2SFCA method. Mao and Nekorchuk [15] first attempted to incorporate multiple transportation modes in spatial accessibility, by dividing each population group into multiple subgroups corresponding to different modes. However, as pointed out by Langford et al. [16], there are some shortcomings in their method. First, Mao and Nekorchuk only yielded a single combined accessibility score, but not separate accessibility scores for each subpopulation. Second, they calculated travel time by private car and by public transit based on the same road network dataset but with different speeds, thus the travel time by bus in their calculation was under-estimated as the bus routes are limited to only a proportion of the roads.

Langford et al... [16] proposed an advanced multi-modal 2SFCA method. They improved Mao and Nekorchuk’s method [15] by estimating travel time by public transit based on the actual bus network. In addition, they also demonstrated accessibility for each population subgroups in the results.

Mao and Nekorchuk’s method [15] is formatted based on the original 2SFCA, while Langford et al’s method [16] on the basis of the Enhanced 2SFCA. As mentioned above, various improvements with respect to distance decay function can be unified by the generalized 2SFCA framework. Therefore, in this study, the multi-modal 2SCFA method is uniformly formatted based on the generalized 2SFCA framework. In this way, the multi-modal 2SCFA method can be more understandable and easier to be extended compared to the versions in the above two studies.

In this study, we consider two transport modes, i.e. the mode by car and by public transit. Let *p* denotes the public transit mode, and *n* denotes the car mode, then the dual-modal 2SFCA can be formatted as follows. First, the accessibility of each population subgroup by each mode (car-mode and transit-mode for short) can be calculated as follows:

3 $$ {A}_{i,p}={\sum}_j\frac{S_jf\left({d}_{ij,p}\right)}{\sum_k\left\{{D}_{k,p}f\left({d}_{kj,p}\right)+{D}_{k,c}f\left({d}_{kj,c}\right)\right\}} $$

4 $$ {A}_{i,c}={\sum}_j\frac{S_jf\left({d}_{ij,c}\right)}{\sum_k\left\{{D}_{k,p}f\left({d}_{kj,p}\right)+{D}_{k,c}f\left({d}_{kj,c}\right)\right\}} $$

where *A*_*i*, *p*_ and *A*_*i*,  *c*_ are the transit-mode accessibility of the car-mode accessibility at demand node *i* respectively. *D*_*k*, *p*_ and *D*_*k*, *c*_ are the transit-mode population and the car-mode population at demand node *k* respectively. *d*_*kj*, *p*_ and *d*_*kj*, *c*_ are the transit-mode and the car-mode travel time from demand node *k* to facility *j* respectively. *f* is the distance decay function, which is the same with formula (2) by simply replacing *d*_*ij*_ by *d*_*ij*, *p*_ or *d*_*ij*, *c*_. The catchment area size *d*_0_ is the same for both modes.

Second, the combined accessibility at each demand node can be calculated as the weighted average of *A*_*i*, *p*_ and *A*_*i*,  *c*_ as follows:

5 $$ {A}_i=\frac{D_{i,p}{A}_{i,p}+{D}_{i,c}{A}_{i,c}}{D_{i,p}+{D}_{i,c}} $$

where *A*_*i*_ is the combined accessibility at demand node *i* and other variables are the same with formula (3) and (4).

### Estimation of travel time

As for the estimation of travel time by each transport mode, various measures have been adopted in existing studies. The travel time measure seems to be the most superior measure of the actual spatial impediment between demanders and facilities [23], which not only reflects the absolute spatial distance between two sites, but also depends on the level of mobility and available transportation modes. Most studies estimate travel time by setting a driving speed for each rank of roads according to technical limitation of roads [24] or actual driving speed in the study area [25], then using network analysis tools to calculate the minimum travel time between any two sites. This estimation method is also adopted by the two abovementioned studies of multi-modal 2SFCA method. Specifically, Mao and Nekorchuk [15] used the same road network dataset to estimate car-mode and transit-mode travel time. Though driving speeds were set as different for each mode, the estimation of transit-mode travel time was inaccurate as the transit routes are limited to only a few roads rather than the whole road network. Langford et al. [16] improved the estimation of transit-mode travel time by using actual bus network dataset. One shortage of this method is that the accuracy of estimation depends on driving speed, which is somewhat arbitrary in practice. Moreover, road network data available to personal researchers are often outdated [17]. Public transit network data is even unavailable to personal researchers in many cases.

Recently, some studies have introduced APIs of online map developers such as Google Map or Baidu Map to estimate travel time [11, 17, 26]. In this way, researchers can make use of the dynamically updated transport network data and the routing rules maintained by map developers to obtain a reliable estimation of travel time [17]. APIs are usually available to personal researchers. This study utilizes Baidu Map API (lbsyun.baidu.com/index.php) by programming in JavaScript to estimate travel time. Two independent APIs, the Transit Searching API and the Driving Searching API, have been developed by Baidu Map to estimate transit-mode and car-mode travel time respectively.

### Parameters

The ratios of car mode and public transit mode are determined according to the car ownership ratio in Shenzhen. It is calculated using the number of private cars and total population from the Shenzhen Statistical Yearbook 2015, and the average household size from the 2010 Population Census of Shenzhen City. The result shows that there are 54 private cars per hundred households in Shenzhen. Therefore, the ratios of car mode and public transit mode are set as 0.54 and 0.46 respectively. The car-mode and transit-mode population at each demand node are calculated by multiplying the total population at each demand node and the ratio of each mode.

**Fig. 3:**
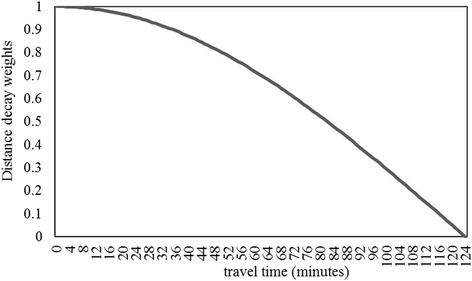
The curve of Gaussian distance decay function

According to travel time calculated above, the maximum travel time via public transit from each sub-district to the nearest hospital is 120 min. The distance decay weight is 1 for zero travel time. Following existing study [11], the catchment area size *d*_0_ is set as 124 min, such that the distance decay weight of the maximum travel time (120 min) is close to zero. In this way, the sub-district that is farthest from hospitals can access healthcare services but with a very small weight (0.05). The curve of the distance decay function is shown in Fig. 3.

## Results

### Results of multi-modal 2SFCA method

**Fig. 4:**
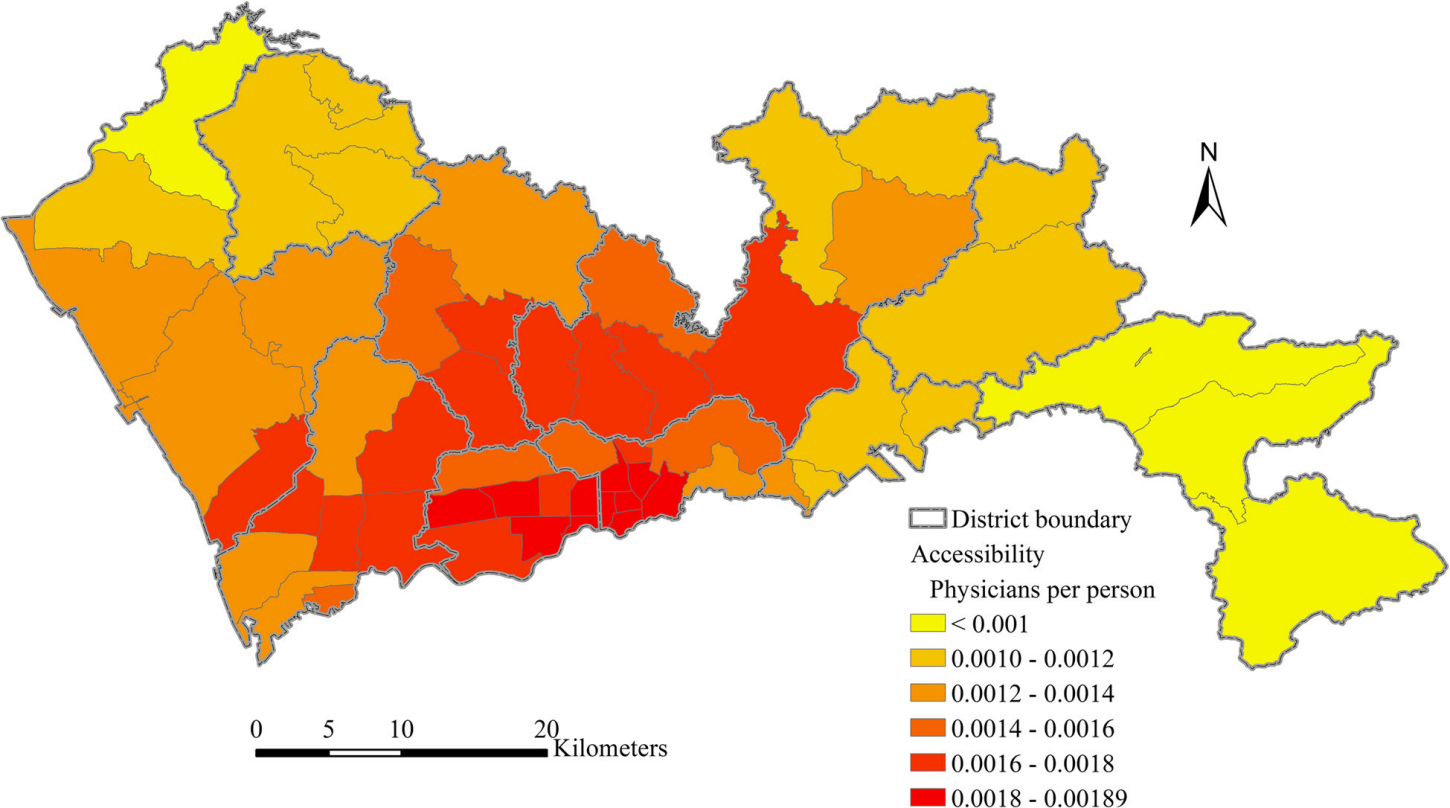
Healthcare accessibility by multi-modal 2SFCA in Shenzhen

The ratio of total physicians to total population in Shenzhen is 0.00144, indicating that there are 1.44 physicians for per thousand persons in average. This total supply-to-demand ratio is equivalent to the population-weighted average accessibility of all sub-districts. According to the result of the multi-modal 2SFCA method, the range of accessibility scores is from 0.00066 to 0.00189. As shown in Fig. 4, the distribution of healthcare accessibility by multi-modal 2SFCA shows significant spatial disparity. The accessibility in the central region is higher than the eastern and western part of Shenzhen. Sub-districts with the highest healthcare accessibility are mainly located in Luohu and Futian, followed by Nanshan, Longhua and western Longgang. These sub-districts have advantages in access to healthcare services because of their central locations. The road network is also more developed in these sub-districts. All sub-districts in Dapeng District and the most northern sub-district in Bao’an District, which are the most peripheral sub-districts in Shenzhen, have the lowest accessibility.

### Comparison between single- and multi-modal accessibility

**Fig. 5:**
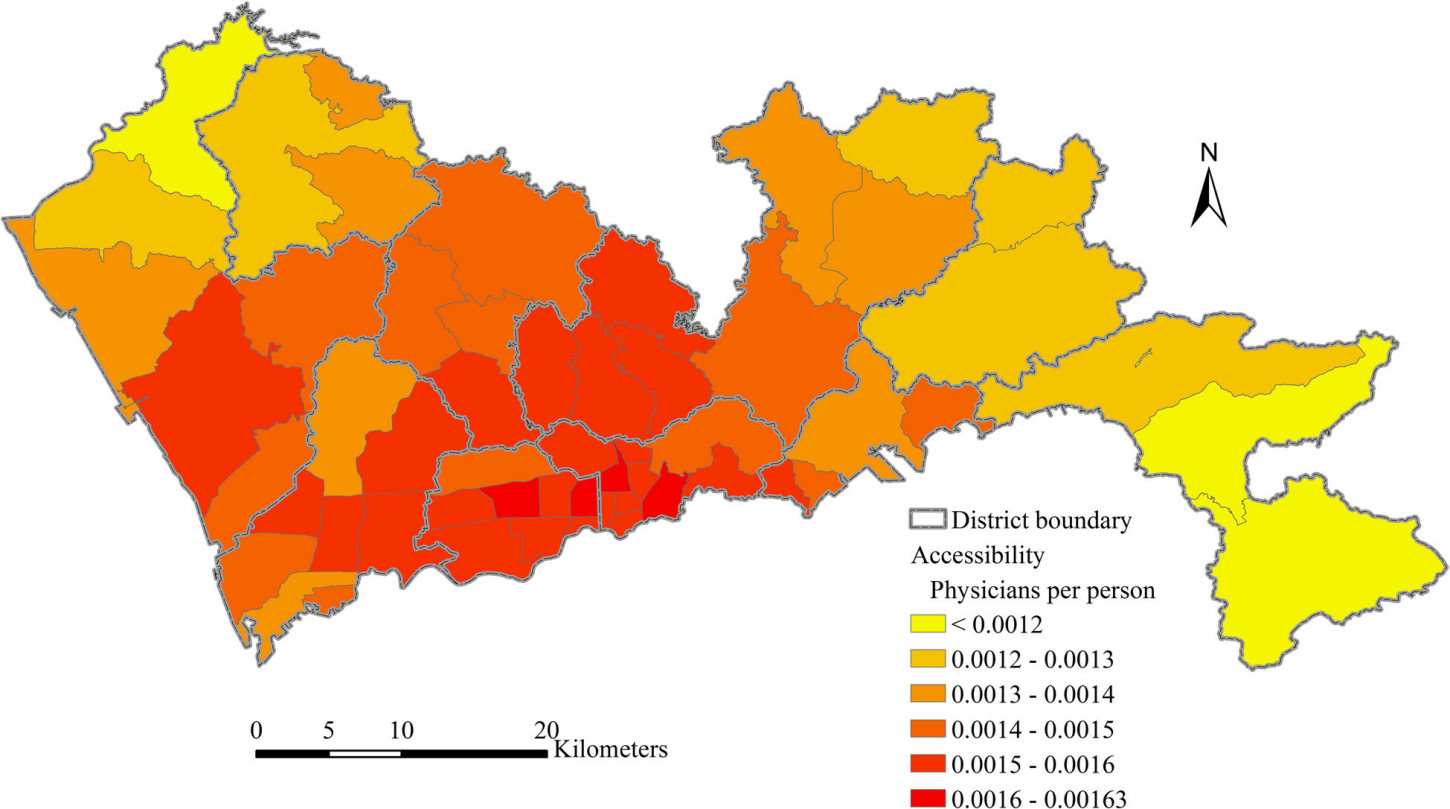
Healthcare accessibility by single-modal 2SFCA in Shenzhen

To compare the multi-modal 2SFCA with the traditional single-modal 2SFCA, the healthcare accessibility is also calculated using the single-modal 2SFCA. Following most applications of single-modal 2SFCA, the travel cost between each demand node and facility is measured by travel time by car. The spatial distribution of healthcare accessibility by single-modal 2SFCA (Fig. 5) shows a similar pattern with the multi-modal accessibility. Accessibility in the central region is higher than the eastern and western Shenzhen. However, the range of the accessibility scores by single-modal 2SFCA is from 0.00086 to 0.00162, which is smaller than the range of multi-modal accessibility. This preliminarily indicates that the disparity of single-modal accessibility is smaller than multi-modal accessibility.

**Fig. 6:**
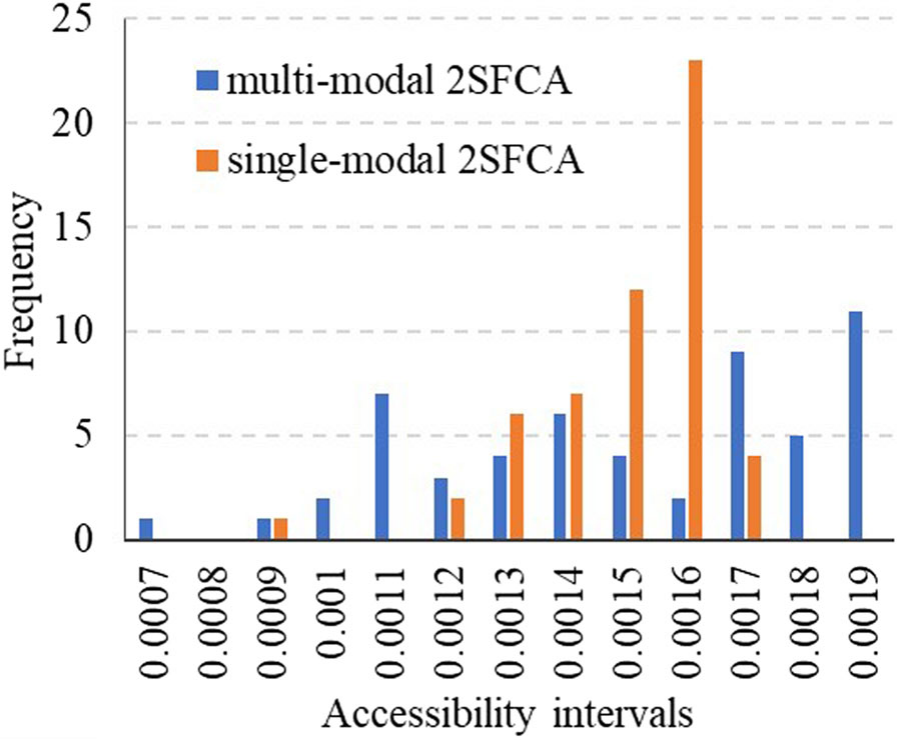
Frequency distributions of accessibility by multi- and single-modal 2SFCA

To conduct a further comparison between the single- and multi-modal method, we plot the frequency distribution of both the multi-modal and single-modal accessibility (Fig. 6). Each scale mark of the horizontal axis denotes an accessibility interval of 0.0001. For example, the first scale mark means ‘accessibility less than 0.0007’, while the second scale mark means ‘accessibility larger than or equal to 0.0007 and less than 0.0008’. The vertical axis is the number of sub-districts lying in each accessibility interval. The frequency distribution of the single-modal accessibility is mainly concentrated between 0.0013 and 0.0016, with 48 out of 55 sub-districts lie in this 0.0004 accessibility interval. By contrast, the frequency distribution of the multi-modal accessibility is much more dispersed and even. This confirms the above conclusion that the disparity of the accessibility by the multi-modal 2SFCA is larger than the traditional single-modal 2SFCA.

### Dividing multi-modal accessibility into subgroups

**Fig. 7:**
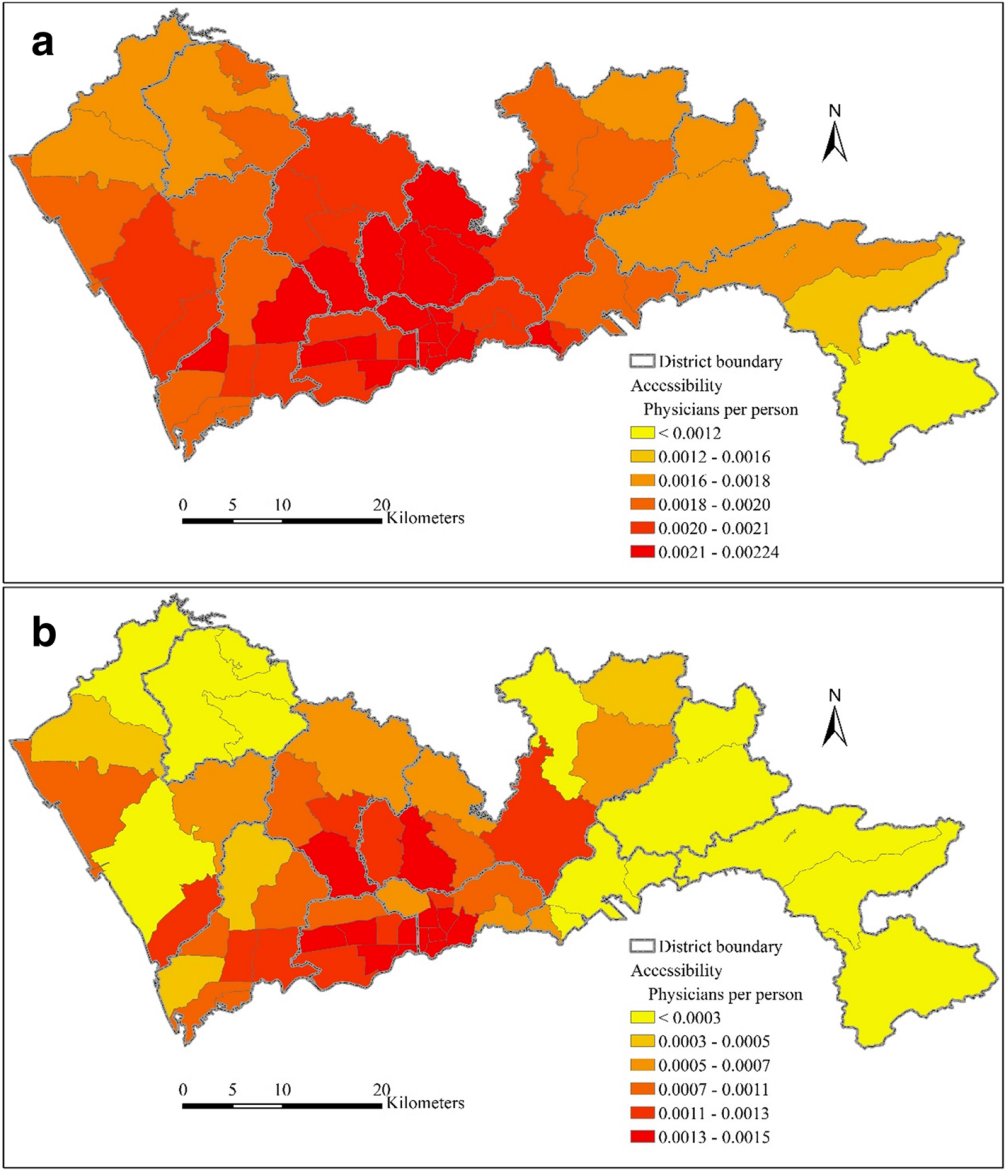
Healthcare accessibility by multi-modal 2SFCA for (**a**) car-mode and (**b**) transit-mode subgroup in Shenzhen

The multi-modal 2SFCA method divides the population at each sub-district into two separate subgroups, one by car mode and one by public transit mode. As expressed by formula (5), the healthcare accessibility for each sub-district shown in Fig. 4 consists of the accessibility of the car-mode subgroup and the transit-mode subgroup. Figure 7 shows the car-mode and transit-mode accessibility obtained by the multi-modal 2SFCA respectively. The population-weighted average of the car-mode and transit-mode accessibility are 0.00199 and 0.00081 respectively. The average car-mode accessibility is 2.47 times of the average transit-mode accessibility.

Figure 7 also shows a significant difference between the spatial distributions of car-mode accessibility and transit-mode accessibility. The spatial distribution of car-mode accessibility is relatively even, though accessibility in the central region is still slightly higher than other regions. By contrast, the spatial distribution of transit-mode accessibility shows a significantly uneven pattern. The transit-mode accessibility in the central region is much higher than the eastern and western Shenzhen. This indicates that it is much easier for the demanders in the central region to access to healthcare services via public transit than demanders in other regions. Based on the comparison of the combined accessibility (Fig. 4), the car-mode accessibility (Fig. 7a) and the transit-mode accessibility (Fig. 7b), it can be concluded that the disparity in accessibility via public transit is the main reason of the uneven pattern of the combined healthcare accessibility in Shenzhen. The public transit network is much more developed in the central region. The impacts of the spatial configuration of healthcare facilities and road network exist, but are relatively smaller.

The coefficient of variation (CV), which is calculated as the ratio of the standard deviation and the mean value, can be used to measure the disparity of accessibility. The value of CV ranges from 0 to 1. A larger CV indicates a larger disparity. In this way, the degree of accessibility disparity in different scenarios can be quantitatively compared. As is shown in Table 1, CV of the multi-modal accessibility is larger than the single-modal accessibility, but both are relatively small. For the accessibility of each subgroup obtained by the multi-modal 2SFCA, CV of the car-mode accessibility is a bit smaller than the single-modal accessibility, while CV of the transit-mode accessibility is much larger than all other scenarios.

**Table 1:** Coefficients of variation of accessibility in different scenarios

Scenario	Multi-modal	Single-modal	Multi-modal (car)	Multi-modal (transit)
CV of accessibility	0.216	0.104	0.102	0.590

## Discussion

According to the comparison, the disparity of the accessibility by the multi-modal 2SFCA is larger than the traditional single-modal 2SFCA. On everage, when dividing multi-modal accessibility into car-mode and transit-mode subgroups, the car-mode accessibility is 2.47 times of the transit-mode accessibility. This indicates that the transit-mode subgroup is disadvantaged in the competition for healthcare services with the car-mode subgroup, for an obvious reason that the travel time by public transit is usually larger than the travel time by car.

The car-mode accessibility and transit-mode accessibility also show significantly different patterns. The spatial distribution of transit-mode accessibility is more uneven, with higher transit-mode accessibility in the central region than the eastern and western Shenzhen. The remarkable disparity of the transit-mode accessibility is the main reason making the disparity of multi-modal accessibility larger than the single-modal accessibility. These conclusions cannot be drawn by the traditional single-modal 2SFCA method.

According to the findings of this study, to reduce the disparity of healthcare accessibility in Shenzhen, a key solution is to improve the public transit conditions for seeking healthcare services. On one hand, public transit routes connecting hospitals and major residential places should be increased. On the other hand, the transfer between public transit (metro or bus) stations and hospitals, by walking or by bicycle, should be emphatically improved. This can be carried out in the short term and at a relatively low cost. Another solution is to optimize the spatial configuration of hospitals. More healthcare service resources (physicians in this study) should be allocated in the eastern and western Shenzhen, especially sub-districts in Dapeng District and western Bao’an District. This can be implemented by constructing new hospitals or expanding the existing hospitals in these regions.

## Conclusions

This study utilized the multi-modal 2SFCA method to measure the healthcare accessibility in Shenzhen, China. The model was constructed based on the generalized 2SFCA framework, which enables the integration of advanced 2SFCA with different distance decay functions. In this way, the model combines the multi-modal 2SFCA model proposed by Mao and Nekorchuk [15] and by Langford et al. [16], and thus facilitates the implications of the multi-modal 2SFCA method. In this study, the Gaussian function was adopted to capture the distance decay effects within the catchment areas. In addition, two Baidu Map APIs were utilized to estimate travel time by public transit or by car respectively. Compared to traditional methods for measuring travel time, this method can provide more reliable estimates of travel time, and is easier to be accessed by individual researchers. This improved the estimation accuracy of travel time via different modes compared to previous applications of the multi-modal 2SFCA method.

There are still some limitations in this study. For example, only the citywide car ownership level was used, due to the lack of car ownership data at a finer scale. If the car ownership data at the district scale or sub-district scale can be obtained in the future, the disparity in proportions of different transport modes among different regions can be captured in the method, which can further improve the accuracy of healthcare accessibility.

## Abbreviations

2SFCA: Two-step floating catchment area
API: Application Programming Interface
CV: The coefficient of variation
